# Effect of Collection Month, Visible Light, and Air Movement on the Attraction of Male *Agriotes obscurus* L. (Coleoptera: Elateridae) Click Beetles to Female Sex Pheromone

**DOI:** 10.3390/insects11110729

**Published:** 2020-10-26

**Authors:** Joyce P. S. Leung, Jenny S. Cory, J. Todd Kabaluk, Alida F. Janmaat

**Affiliations:** 1 Department of Biological Sciences, Simon Fraser University, Burnaby, BC V5A 1S6, Canada; joycepsleung@gmail.com (J.P.S.L.); jsc21@sfu.ca (J.S.C.); 2 Agriculture and Agri-Food, Agassiz, BC V0M 1A2, Canada; Todd.Kabaluk@canada.ca; 3 Biology Department, University of the Fraser Valley, 33844 King Rd, Abbotsford, BC V2S 7M8, Canada

**Keywords:** chemical ecology, attraction distance, environmental factors, behavior, pest management

## Abstract

**Simple Summary:**

Wireworms are larvae of adult click beetles and can be major pests of many crops. The larvae live for several years in the soil and are difficult to manage, so additional control methods are being investigated, including the targeting of their adult stage, click beetles. For example, sex pheromones of female beetles can be used to attract males to a substrate treated with an insecticide. We examined whether the response of male click beetles to female sex pheromone is affected by environmental and seasonal factors i.e., beetles collected from the field in different months. Using small-scale lab experiments, we found that the beetles’ response to pheromone was not affected by light, but that air movement made them move faster. Exposure to pheromone made the beetles move more, but this did not vary with collection month, although beetles collected in May moved more slowly than those collected in March and April. In the field, male beetles were attracted up to 14 m from a pheromone source, the furthest distance tested. Understanding how beetle response to pheromone varies with these factors is important for the refinement of programs aimed at their management.

**Abstract:**

Elaterid female sex pheromone, while currently used for monitoring the adult life stage (click beetle), has only recently been explored as a potential management tool. Consequently, there is little understanding of how abiotic and biotic conditions influence the response of click beetles to the pheromone. We examined whether the response of male *Agriotes obscurus* L. (Coleoptera: Elateridae) beetles to a cellulose-based formulation of female sex pheromone (‘pheromone granules’) is influenced by air movement, presence of visible light, and month of beetle collection. In addition, we investigated the distance from which beetles were attracted to the pheromone granules. Click beetle response was determined by measuring movement parameters in free-walking arena experiments. The response to pheromone was not affected by the presence or absence of visible light. We found that beetles collected earlier in the season had increased activity and interaction with pheromone under moving air conditions, compared to beetles collected later. When controlling for storage time, we confirmed that individuals collected in May were less active than beetles collected in March and April. In the field, beetles were recaptured from up to 14 m away from a pheromone granule source, with over 50% being recovered within 4.4 h from a distance of 7 m or less. Understanding how abiotic and biotic factors affect pest response to pheromone can lead to more effective and novel uses of pheromone-based management strategies.

## 1. Introduction

Wireworms, the larval stage of click beetles (Coleoptera: Elateridae), are important pests in Europe, North America, and Asia [1,2]. They attack underground plant tissue of a range of crops, including potatoes, carrots, rutabaga, and sugar beets, making the crops unmarketable. Wireworms are able to transition through up to 13 instars in the soil over a 30-month period (semi-field conditions), with faster or slower development being temperature dependent [3]. They pupate in late summer, and overwinter as adults which emerge from the soil in the spring and reproduce and die over a few months [4]. Mating can start soon after emergence with peak oviposition occurring between early May and early June [3]. Their subterranean nature makes them difficult to target, with control products exploiting wireworm attraction to carbon dioxide [5] by placing the products close to respiring plant roots, or formulating active ingredients with a CO_2_-producing substrate, e.g., [6]. Once considered a minor pest in North America and Europe [7], their impact has become widespread and problematic, largely due to the deregistration of persistent and toxic chemical insecticides [8]. Changes in agricultural practices, including the adoption of no-till regimes, may have also contributed to the rise of wireworms, by sustaining habitat for beetle reproduction and food for larvae [9]. One of the current options for wireworm management is to use a neonicotinoid seed treatment. However, this class of chemicals only makes the wireworms lethargic and unable to feed, and does not cause mortality. Therefore, they are ineffective in reducing a wireworm population [10,11]. Consequently, there is considerable interest in developing alternative forms of control. Targeting the adult beetles instead of, or in addition to, larvae may reduce reproduction and population growth.

In British Columbia, Canada, *Agriotes obscurus* L. is one of two European species that are considered economically damaging to potatoes [12]. Adult *A. obscurus* beetles eclose in winter and early spring, and emerge from the soil when temperatures are favorable [13], after which mating and oviposition occur. Ovipositing adults are often most numerous in pastures and cereal crops, therefore controlling adult populations is likely to be most effective in these habitats [14]. Targeting the adults in conjunction with the larvae is important, as multiple generations of wireworms may coexist at a given time. In addition, adults are the main dispersive stage of elaterids [15]. In a study by Schallhart et al. [16], *A. obscurus* beetles were found to be capable of dispersing up to 80 m, which was the maximum distance tested. Flight has also been reported in this species in some parts of Europe [17,18]. North American populations have rarely been reported to fly [1,19], although a recent report suggests that mass flight may occur under certain conditions [10].

The female sex pheromones of most major European click beetle pest species, including *A. obscurus,* have been identified [20]. They are mostly used for monitoring purposes [21]; however, a number of pheromone-based management tactics that target adults have been attempted in the past, including mass trapping [22], mating disruption [22,23], and attract-and-kill [24]. Effective lure placement in pheromone-based control strategies requires an understanding of how a pest species responds under different biotic and abiotic conditions [25]. For example, environmental cues may provide information for orientation towards chemical cues. Although chemical gradients can provide information, other sources of directional information, including celestial cues and water and wind currents, may be necessary for successful location of odor sources [26]. Wind speed and direction have been identified as important in determining pheromone trap success in pests, including lepidopterans [27] and boll weevils [28]. Insects may also exhibit search rhythms which can restrict pheromone response to certain periods during the day or year. In most noctuid moths, for example, males respond to pheromone in the mid- to late scotophase, which corresponds to periods of pheromone emission by females. This phenomenon may be mediated or impacted by environmental cues, such as photoperiod (e.g., [29]), temperature (e.g., [30]), or endogenous mechanisms, such as circadian rhythms in pheromone reception (e.g., [29]) or hormonal variation [31]. However, there is limited information on the sensory ecology of *A. obscurus* and other click beetles.

Recently, a granular formulation of female sex pheromone was developed and investigated for use in an attract-and-kill strategy with the fungal pathogen *Metarhizium brunneum* Petch (Ascomycota: Hypocreales: Clavicipitaceae) [24]. A granular formulation of pheromone may prove to be a cost-effective alternative to current liquid formulations and allow for novel application methods. Although the range of attraction for a point source of pheromone has been determined previously [32,33], the attraction distance for a granular formulation is not known. The objectives of this study were to evaluate male *A. obscurus* click beetle response to pheromone under different abiotic conditions under lab conditions, and to determine the attraction range of pheromone to male beetles in the field. We tested whether air movement and presence of visible light affect the response of male *A. obscurus* beetles to the pheromone. We also examined how timing of collection (e.g., March, April, or May) affected male *A. obscurus* response to the pheromone. Understanding how these factors affect pheromone response is vital to facilitating the development of pheromone-based techniques in the management of *A. obscurus* populations.

## 2. Materials and Methods

### 2.1. Beetle Collection and Pheromone Formulation

As rearing click beetles in the lab is time-consuming, we used field-collected beetles. Male *A. obscurus* beetles were collected from research fields at Agriculture and Agri-Food Canada’s Agassiz Research and Development Centre in British Columbia, Canada (Lat 49.243° N, Lon −121.760°) in April and May in 2014, and March, April, and May in 2015, using pitfall traps baited with pheromone. Beetles collected in 2014 were used in experiments 1 and 2, whereas beetles collected in 2015 were used in experiments 3 and 4. In 2014, beetles collected earlier were also stored for longer before being used in the experiments, whereas in 2015, storage time was kept constant. It is not possible to know the age of the field-collected beetles, although all adult beetles die after one season [4]. Unless otherwise stated, the beetles were kept separately, based on when they were collected, in groups of no larger than 300 beetles, at 10 °C in darkness. Beetles were kept at this temperature to delay natural physiological progression [34]. Average seasonal temperatures were: March: 3.4/11.6 °C; April: 5.9/15.5 °C; May: 8.9/18.9 °C. Beetles were fed organic apple pieces ad libitum, and provided with fresh forage grass clippings (*Poaceae* spp.) for cover until use. All beetles were verified as *A. obscurus* when selecting them for experiments (occasional contaminant species was *Agriotes lineatus* L.). The pheromone source was created in our lab by impregnating a cellulose-based granule with a synthetic blend of *A. obscurus* pheromone (1:1 blend of geranyl octanoate (also known as geranyl caprylate) and geranyl hexanoate (also known as geranyl caproate); Penta Manufacturing Company, New Jersey) in a proprietary process (1% wt/wt, Scotts Canada, Delta, BC, Canada). Non-impregnated cellulose granules (‘blank’) were used as controls.

### 2.2. Experiment 1: Air Movement

**Figure 1:**
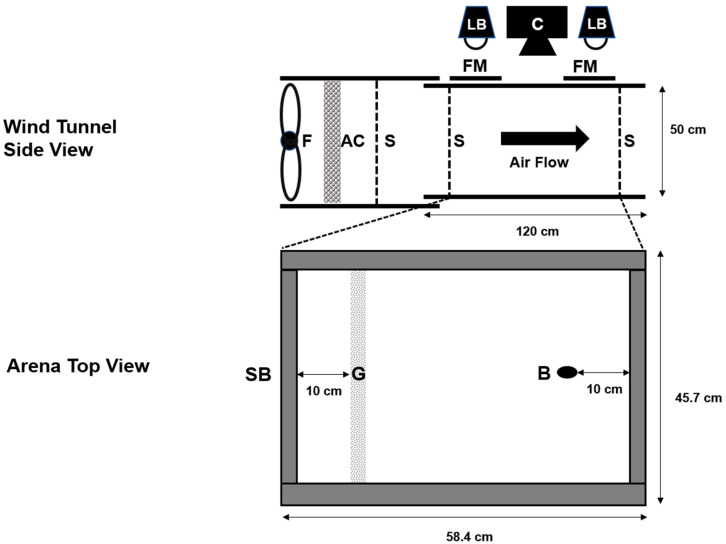
Wind tunnel arena. Tunnel was constructed using 4-mm-thick Plexiglass. Corrugated plastic framed screens (S) were used to section off the middle to form an arena, the base of which was lined with brown dry sheathing paper, which was held down by 2-cm-wide, 3-mm-thick steel bars (SB) on all four sides. A band of blank or pheromone granules (G) were introduced 10 cm from the upwind end of the arena, and test beetles (B) were placed 10 cm from the downwind end of the tunnel at the start of trials. The tunnel was illuminated with two 23-W compact fluorescent lamp blubs (Daylight Mini Twister, Philips Lighting, Markham, ON, Canada) located on both sides of the arena (LB), which were diffused with a layer of foam (FM). At one end of the tunnel, a 52 cm × 52 cm box fan (F) was used to drive wind through activated charcoal (AC).

Here, we tested whether air movement affects the response of *A. obscurus* males to pheromone using a wind tunnel arena. Behavioral assays took place throughout July and August in 2014, between 10:00 and 18:00 h at room temperature (25–27 °C), across 17 days. Beetles were exposed to either pheromone or blank granules under moving (1.2 m s^−1^) and still air conditions in a full factorial design. The wind speed was consistent with natural wind speeds during the months of April, May, and June at the Agassiz Research and Development Centre from 2010 to 2013, which were 1.5 m s^−1^ ± 0.6 (day) and 1.0 m s^−1^ ± 0.3 (night) [35]. Beetles were removed from cold storage 2–4 h before testing. Their behavior was assessed qualitatively and appeared to be normal. A single beetle was placed into a wind tunnel (Figure 1), 10 cm from the downwind end of the tunnel, and covered with a 1 oz (29.6 mL) plastic cup (Solo Cup Company, Lake Forest, IL) for 5 min to acclimatize. A 2-cm-wide band of granules (0.9 g) was applied 10 cm upwind using a stencil. The beetle was video-recorded for 10 min with a GigE camera (Basler acA1300-60gc, Ahrensburg, Germany). Beetles were only used once. Beetles that did not move in the initial three minutes, or climbed onto the screens at either end, were discarded from the analysis. Out of the 211 beetles tested, 42 were discarded. A total of 19–23 replicates were conducted for each of the four air movement–pheromone combinations for each month of beetle collection (April: N = 87; May: N = 82).

Videos were analyzed using Ethovision XT 10 software (Noldus, Wageningen, The Netherlands). Videos were sampled at 8.9 frames per second, and tracks were visually inspected and corrected manually by relocating points to the beetle where the software incorrectly identified parts of the arena as the target beetle. A lowess smoothing algorithm was applied afterwards, to reduce noise in tracking. Beetle walking speed, distance moved, time to first contact with the pheromone band (latency of response), the number of contacts, and duration of the contacts with the granules were used as measures of the beetles’ response to pheromone. In experiments 1 and 2, collection month was confounded with storage time, such that beetles that were collected earlier in the season were stored for a longer period of time, making these two factors inseparable. A three-way, linear mixed model was conducted with date of collection (April, May), pheromone treatment (pheromone, blank) and air movement (moving, still) included as fixed effects (Table 1), with date included as a random effect. To further examine the significant three-way interaction, a two-way linear mixed model was conducted separately for the April and May beetles. The frequency and duration of contacts with pheromone granules were square root transformed and natural log transformed, respectively, to normalize residuals.

### 2.3. Experiment 2: Visible Light

In this experiment, beetle response to pheromone was measured in simulated daylight and darkness. Traditionally, darkness has been simulated by using red light. However, some insects, such as *Anopheles gambiae* Giles (Diptera: Culicidae) mosquitos and *Pterostichus melanarius* Illiger (Coleopatera: Carabidae) ground beetles, have been shown to sense red light [36,37]. The spectral sensitivity of click beetles is unknown, therefore the treatments are referred to as visible light treatments. The effect of visible light on the response of *A. obscurus* males to pheromone was carried out in circular arenas made from open-top Petri dishes (140 mm × 15 mm) lined with paper towel, which was replaced after each test. Behavioral assays took place in August 2014 over 6 days. The arena was illuminated from a single white or red compact fluorescent light bulb placed directly overhead (white: Energy Saver Twister 23 W Daylight Deluxe, red: Energy Saver Mini Twister Red 13 W, Philips, Markham, ON, Canada). This created a difference in illuminance of 0.8 Klux and 0 lux, respectively. Ambient lighting was turned off during the experiment. A single beetle was placed in a 2-cm diameter acetate ring; after 3 min, 0.03 g of pheromone-impregnated or blank granules were introduced into another acetate ring placed in the center of the arena. After the rings were removed, the beetle was video-recorded for 10 min. A total of 9–12 beetles were tested for each light-pheromone treatment and month of beetle collection combination across six days.

Walking speed, distance walked, and granule contact frequency and duration were analyzed as in Experiment 1, except that the third factor was light type instead of air movement. Only beetles that had reached the granule zone were analyzed for frequency and duration of contacts with granule zone. The probability of reaching the granule zone was analyzed as a logistic regression. The frequency and duration of contacts with granules were square root transformed and natural log transformed, respectively, to normalize residuals (Table 1). The proportion of time spent moving was square transformed and analyzed with a generalized linear mixed model with beta distribution and logit link function.

### 2.4. Experiment 3: Month of Beetle Collection

In the previous two experiments, we found that beetles collected from different months had different levels of activity, suggesting that the timing of beetle collection might be an important factor in determining beetle response. As storage time was not controlled in the previous two experiments (i.e., beetles collected earlier would have been stored for longer), we conducted a separate experiment to investigate the effect of when the beetles were collected. Movement behavior of beetles collected in March, April, and May 2015 were compared with, and without, pheromone (i.e., 2 × 3 design). A group of beetles from each month was tested post-collection, after a short period over two days (17 or 18 days) or after a longer period (38 or 39 days). Beetles were stored at 23 °C, 12 h L:D, to allow the beetles’ natural physiological progression to continue. Test arenas (replicates) were paper-lined Petri dishes (90 mm × 15 mm), covered in a mesh lid, that were placed in a group of five around a central pheromone source. A single beetle was placed into each dish and allowed to acclimatize for 3 min, after which 0.75 g of pheromone granules or blank granules were introduced into a central 1 oz cup trimmed to 1 cm high. Beetles were recorded for 11 min. Walking speed, distance walked, and proportion of time spent moving were measured. Each beetle was used only once, and five to seven replicates (i.e., of the five Petri dish set-ups) were used per pheromone/collection month/storage time combination, resulting in 88–110 beetles tested per pheromone/collection month treatment (Supplementary Table S4). Beetles that did not move were discarded, resulting in 1–5 valid beetles per replicate. Videos were analyzed as previously described. Experiment 3 was analyzed with a linear mixed model, where month of beetle collection and pheromone were included as fixed effects, and test dates were included as a discrete random effect (Table 1). The group mean of a replicate was used as the response variable, weighted by the number of beetles per replicate.

### 2.5. Experiment 4: Beetle Response Range

We examined the distance over which the beetles were attracted to the pheromone granules in a mark–release–recapture experiment in a grass-clover forage field near Agassiz, British Columbia, Canada. Eight 30 × 3 m plots, spaced 20 m apart, were marked out, and the grass mowed to 10 cm in height (grass height outside of plots was left at ~50 cm). Plots were oriented lengthwise in a north–south direction, with the south side being close to the perimeter of the field. Four plots had pheromone granules applied, while the other four had blank granules applied as a control. Pheromone treatment was assigned to each plot in randomized blocks, where two adjacent plots were considered a block. A band of 1.6 g of pheromone granules was introduced across the middle of the plot. Organic granules were used to comply with organic certification requirements of the farm. The initial pheromone release rate of an analogous sample of granules indoors was 278 μg per day, which then decreased to a steady rate of 20–40 μg per day at four days post-deployment [38].

Beetles were marked with enamel model paint (Testor’s Enamel Paint, Rockford, IL, USA) to indicate plot, release distance, and release side. Two sets of 16–18 beetles were released at 1, 3, 7, and 14 m on both sides of the band. Beetles were recaptured with unbaited pitfall traps made from plastic cups (⌀ = 7 cm) placed in the middle and on both ends of the granule band, (i.e., three traps per plot). Pitfall traps were checked after 1 h, and then every 3.5 h up to 13 h. Traps were checked every 24 h thereafter, for a total of six days.

The effects of pheromone granule treatment, release distance, and release side on the proportion of beetles recovered were analyzed using a generalized linear mixed model with a binomial distribution and logit link function (Table 1). Release distance, release side, and treatment were included as fixed effects, with release distance treated as a continuous variable. Plot was included as a random effect. In all statistical models, non-significant interactions were removed first, followed by non-significant main effects that were not part of a significant interaction. All experiments were analyzed using SAS University Edition (SAS Institute, Cary, NC, USA).

## 3. Results

### 3.1. Experiment 1: Air Movement

Most of the activity measures were influenced by a three-way interaction among month of beetle collection, air movement, and pheromone presence (walking speed: *F*_1,145_ = 10.0, *p* = 0.002; distance walked: *F*_1,145_ = 10.38, *p* = 0.002; number of contacts with granule band: *F*_1,145_ = 5.76, *p* = 0.018), therefore we reanalyzed the data separately by collection date.

**Figure 2:**
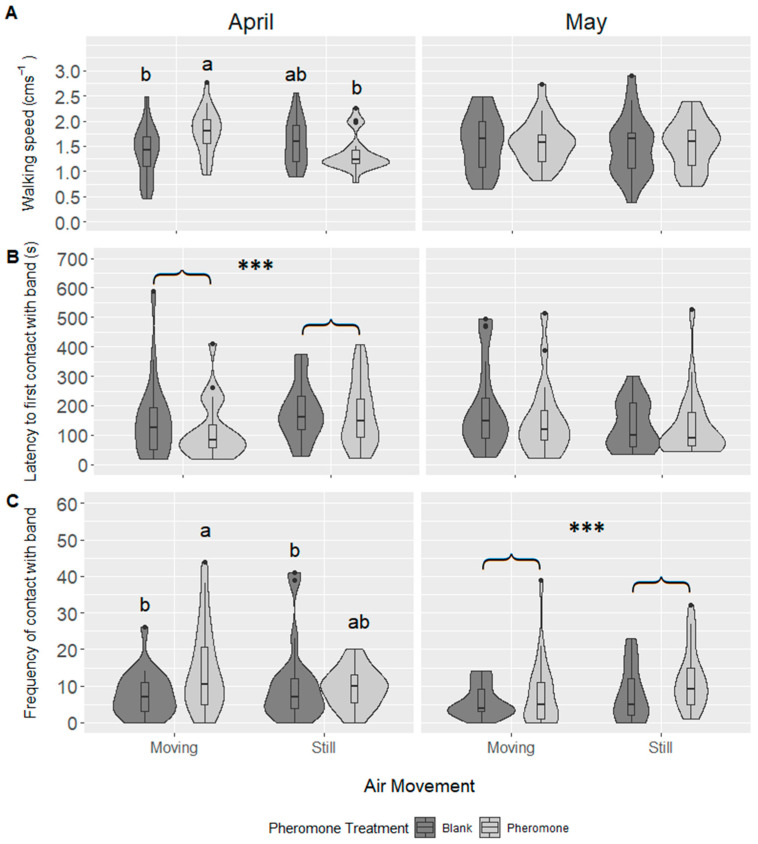
Impact of pheromone and air movement on male *Agriotes obscurus* click beetles. (**A**) LS mean (±SE) walking speed; (**B**) time to first contact with pheromone band; and (**C**) frequency of contacts with band. April: N = 87; May: N = 82. Either side of the violin plot shows the distribution of the data via a probability density, with wider sections indicating values occurring at higher frequency. *** represent significance *p* < 0.05, as assessed by contrasts in a generalized linear model. Letters represent significance at *p* < 0.05 (Tukey’s HSD).

Generally, beetles collected in April, which would have been stored for longer, were more influenced by air movement than those collected in May. For the April beetles, walking speed increased in the presence of pheromone in moving air (pheromone × air movement: *F*_1,68_ = 17.92, *p* < 0.001; pheromone: *F*_1,68_ = 1.58, *p* = 0.212; air movement: *F*_1,68_ = 3.98, *p* = 0.05, Figure 2A), but this effect was not seen in beetles collected in May (pheromone: *F*_1,63_ = 0.00, *p* = 0.961; air movement: *F*_1,63_ = 0.11, *p* = 0.744; pheromone × air movement: *F*_1,62_ = 0.30, *p* = 0.587). The distance walked by the beetles followed the same pattern (April: pheromone × air movement: *F*_1,68_ = 17.8 *p* < 0.001: pheromone: *F*_1,68_ = 17.8, *p* < 0.001; air movement: *F*_1,68_ = 17.8, *p* < 0.001: May: *F*_1,63_ = 0.03, *p* = 0.852; air movement: *F*_1,63_ = 0.00, *p* = 0.974, pheromone × air movement: *F*_1,62_ = 0.47, *p* = 0.498).

In April, moving air increased the response time by 1.5 times, but pheromone had no effect (April: pheromone × air movement: *F*_1,62_ = 0.32, *p* = 0.575; pheromone: *F*_1,63_ = 4.97, *p* = 0.424; air movement: *F*_1,64_ = 5.11, *p* = 0.027, Figure 2B). Again, there was no difference in response in the beetles collected in May (pheromone × air movement: *F*_1,55_ = 0.02, *p* = 0.896; pheromone: *F*_1,57_ = 2.06, *p* = 0.156; air movement: *F*_1,56_ = 0.28, *p* = 0.598). Air movement increased the number of contacts with the band when pheromone was present in April beetles (pheromone × air movement: *F*_1,68_ = 4.84, *p* = 0.031; pheromone: *F*_1,68_ = 6.49, *p* = 0.013; air movement: *F*_1,68_ = 2.21, *p* = 0.1416, Figure 2C), but neither treatment influenced the total time spent in the band (pheromone × air movement: *F*_1,68_ = 1.18, *p* = 0.281; air movement: *F*_1,68_ = 2.83, *p* = 0.097; pheromone: *F*_1,68_ = 3.25, *p* = 0.076). However, beetles collected in May made more contacts with the band and spent longer in the band when they were in still air (contacts: *F*_1,64_ = 5.03, *p* = 0.028; duration: *F*_1,64_ = 5.55 and *p* = 0.022, Figure 2C), and the pheromone had no effect (contacts: pheromone: *F*_1,63_ = 1.05, *p* = 0.309; pheromone × air movement: *F*_1,62_ = 1.54, *p* = 0.220: duration: pheromone: *F*_1,63_ = 1.43, *p* = 0.237; pheromone × air movement: *F*_1,62_ = 2.24, *p* = 0.140).

### 3.2. Experiment 2: Visible Light

The presence of visible light had no impact on any movement parameter, nor did it affect the response to pheromone (Supplementary Information 1 and 2). As expected, beetles exposed to pheromone walked 72% faster and 31% farther than beetles in the blank control (speed: *F*_1,67_ = 27.0, *p* =< 0.001; distance: *F*_1,67_ = 28.52, *p* < 0.001), and also spent a greater proportion of time moving (*F*_1,68_ = 4.92, *p* = 0.030). In the pheromone treatment, 24% more beetles reached the granule zone, compared with the blank control (Wald χ^2^ = 8.98, *p* = 0.003). Of those beetles that reached the granule zone, individuals exposed to pheromone had 75.9% more contacts, compared to those in the blank treatment (*F*_1,46_ = 6.49, *p* = 0.014). However, pheromone did not increase the duration of contact with the granule zone (*F*_1,45_ = 0.56, *p* = 0.457).

Beetles collected in April walked faster and farther than beetles collected in May (walking speed: *F*_1,67_ = 6.24, *p* = 0.015; distance: *F*_1,67_ = 5.62, *p* = 0.025), but there was no difference in how long they spent walking (*F*_1,67_ = 0.58, *p* = 0.449) (Supplementary Information 3). Beetles collected in May spent 85.1% more time in the granule zone, compared to those collected in April (*F*_1,46_ = 7.47, *p* = 0.009).

### 3.3. Experiment 3: Month of Beetle Collection

**Figure 3:**
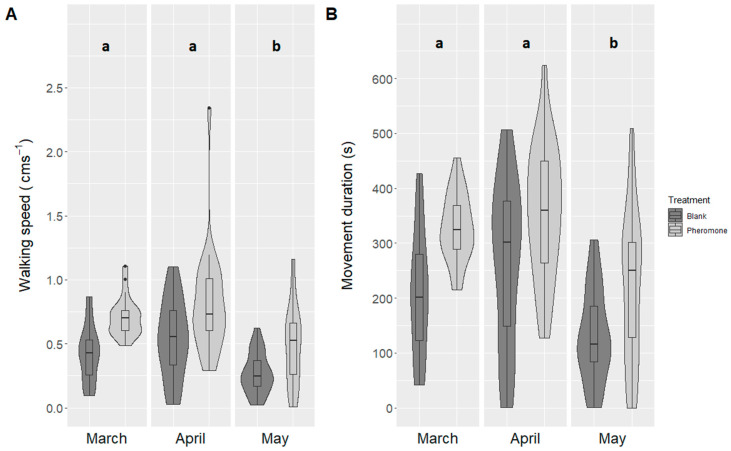
Impact of collection month on male *Agriotes obscurus* click beetle activity in response to pheromone treatment for beetles. Total beetles tested (number of batches with 1–5 beetles in a batch/replicate): March: 220 (44); April: 188 (47); May: 195 (48). Activity is depicted by mean (±SE) (**A**) walking speed and (**B**) movement duration. Letters represent significance at *p* < 0.05, as assessed by Tukey’s HSD.

The month during which the beetles were collected affected their activity, but not their response to pheromone (Table 2, Figure 3).

Beetles collected in March and April did not differ in walking speed, distance moved, or the time that they spent moving; however, beetles collected in May walked more slowly and not as far, compared to those collected earlier. As predicted, beetles in the pheromone treatment walked faster, farther, and longer than beetles without exposure (Table 2, Figure 3).

### 3.4. Experiment 4: Beetle Response Range

**Figure 4:**
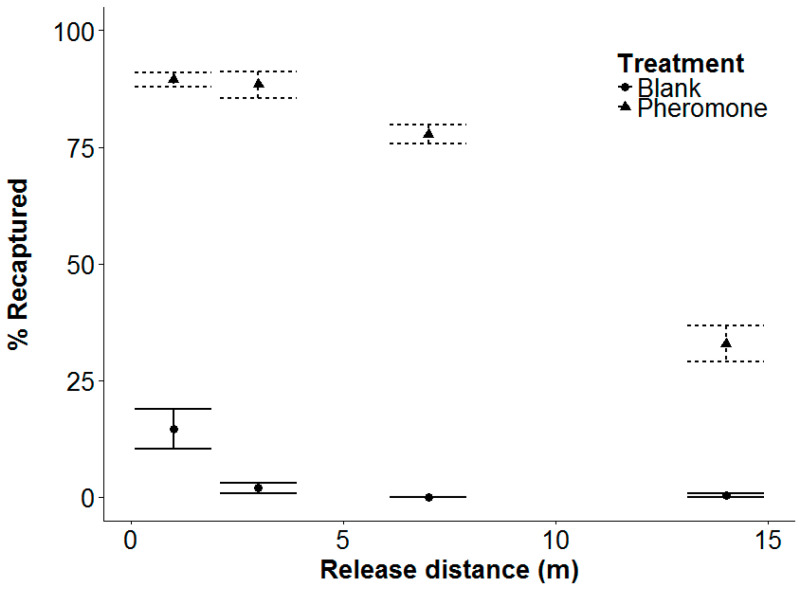
Mean proportion (±SE) of *Agriotes obscurus* beetles recovered after release from 1, 3, 7, and 14 m away from a band of blank or pheromone granules. N = 4 per treatment. The final model is % recovered = 4.0045 (pheromone) − 0.693 (distance) + 0.4396 (release side) +/− 0.4513(distance × pheromone) − 1.480.

In the field trial, a much higher proportion of beetles (72.2% ± SE 1.7) were recaptured in the pheromone treatment, compared to the blank control (4.3% ± SE 1.4). The number of beetles recaptured decreased with the release distance, but the rate of decrease was greater for the blank treatment (pheromone × release distance: *F*_1,53_ = 9.7, *p* = 0.003, Figure 4). For the pheromone treatment, over 50% of beetles were recovered within 4.4 h (SE 0.88) for distances up to 7 m. Mean time to maximum recaptures at each release distance in the pheromone plots are shown in Table 3. More beetles were recaptured from the south than the north (*F*_1,53_ = 9.32, *p* = 0.004).

## 4. Discussion

Overall, we found that only air movement affected the response of *A. obscurus* males to pheromone, but the presence of visible light and the timing of the collection month did not. However, the timing of the collection month did affect the activity of the beetles, with decreased activity in beetles collected later in the spring.

### 4.1. Male Click Beetles Respond to Pheromone under Both Moving and Still Air Conditions

The importance of air currents as directional cues for odor reception has been most emphasized in lepidopterans, although walking insects can also use wind as a directional cue (e.g., silk moth *Bombyx mori* L. (Lepidoptera: Bombycidae) [39]; American cockroach *Periplaneta Americana* L. (Blattodea: Blattidae) [40]; flour beetle *Tribolium castaneum* Herbst (Coleoptera: Tenebrionidae) [41]). Our data suggest that air movement enhances the ability of click beetles to locate and respond to pheromone, with beetles that were collected earlier in the season responding more strongly. Click beetles exhibit strong thigmotactic behavior. In rectangular arenas, we observed that click beetles spent a large proportion of time in the corners, compared to circular arenas, which could have decreased our ability to detect changes in activity in our first experiment. Our results partially corroborate a recent study [42] that found that air movement similar to that of our study (2 m s^−1^) enhanced the response range of beetles to pheromone (at a 6 m distance), whereas at higher wind speeds (6 m s^−1^), beetles were deterred. In our study, air movement did not decrease the response time to pheromone; however, this may have been due to the size of the experimental arena. The observation that click beetles use air movement to orient towards a pheromone source is consistent with the behavior of click beetles in nature. In the field, we observed that males in the presence of female sex pheromone often climb to the top of grass blades [24]. This behavior is likely performed so that beetles can position themselves to sense air movement cues. On the ground, air movement may not provide a reliable directional cue, as micro habitat features can “stir and dilute” pheromone plumes [43].

### 4.2. Visible Light Shows No Impact on Pheromone Response

*A. obscurus* has been reported to exhibit diel patterns of activity and quiescence [44]. It has also been suggested that click beetles are negatively phototropic [13]. Blackshaw et al. [42] observed that the proximity of released *A. obscurus* to a window, which was an untested factor in their experiment, had an effect on beetle response to pheromone. We therefore expected that the presence of visible light could influence beetle response to pheromone. Kabaluk (unpublished data) observed an oscillating pattern of recapture of *A. obscurus* and *A. lineatus* beetles in pheromone-baited pitfall traps over a 24-h period. Beetles were released into an outdoor 2-m diameter enclosure. Hourly assessment of traps revealed peak recapture during three times: 1 a.m., 10 a.m., and 8 p.m., seemingly negating a difference in the response of beetles to pheromone under light. Although the presence of visible light in our study did not have an effect in either the presence or absence of pheromone, this does not rule out other factors that signal time of day influencing male beetle response to pheromone as suggested in other studies, such as temperature or humidity [13], conditions that were not present in our lab-based study.

### 4.3. Male Click Beetles More Active in March and April

Pheromone trapping of beetles showed a capture pattern of low numbers early in the season, with a peak occurring during the first two weeks of May. Our experience with pheromone trapping over a ten-year period indicates that emergence can begin as early as late March, with activity continuing into July, with peak activity generally occurring between mid–late April to late May, depending upon weather. Our experiments indicate that beetles collected earlier in the spring had a higher level of activity than those collected later. This was evident, even when we controlled for the amount of time the beetles were stored prior to use in the bioassays. The effect of beetle collection timing can be due to environmental effects, prior mating experience in the field, and beetle senescence. Although we did not control for temperature or humidity in our experiments, we contend that changes in activity level were not due to environmental factors, as room temperatures did not vary greatly (25–27 °C). It is more likely that effects of beetle collection timing are due to beetle senescence. Although it is not possible to determine the age of beetles collected in the field, beetles emerge in the field over a few months in the spring and early summer. The mean age of beetles captured on one day will be different to those collected at another time, although the degree to which cohorts overlap is unknown. Still, aging in insects can lead to the degeneration of the nervous system as well as the musculoskeletal system, which can lead to a decline in spontaneous locomotion. Decline of locomotor activity due to age has been reported in *Blaberus discoidalis* Audinet-Serville (Blattodea: Blaberidae) cockroaches [45], *Drosophila melanogaster* Meigen (Diptera: Drosophilidae) vinegar flies [46], and *Apis mellifera* L. (Hymenoptera: Apidae) honey bees [47]. This is further corroborated by our observations that beetles collected in May contacted the granule band more in still air, compared to moving air, while the same effect was not seen in beetles collected in April; a deterioration of the musculoskeletal system is consistent with the inability to withstand a wind speed of 1 m s^−1^. Flying insects have been found to be inhibited by upwind movements when high wind speeds are encountered, regardless of whether pheromone is present or not (e.g., *Macrosiphum euphorbiae* Thomas (Hemiptera: Aphididae) potato aphid [48]). This is observable in walking insects too; Hardee et al. [49], for example, found *Anthomis grandis* Boheman (Coleoptera: Curculionidae) boll weevils were deterred by winds greater than 7 km h^−1^.

Although we found changes in overall activity with the timing of beetle collection, we did not find that beetle collection period affected the response to pheromone. In our beetle collection period experiment, although beetles had reduced activity later in the season, they consistently walked twice as fast in the pheromone treatment, although the interaction between arena size and pheromone response was not tested. Insect age has been found to be a determining factor in pheromone response [50,51], and this should be considered in management strategies that use pheromone. In particular, we can reasonably expect our observations to affect the attractiveness of a pheromone lure throughout the season.

### 4.4. Beetle Response Range and Recapture Rates

The cellulose-based formulation of pheromone attracted male beetles from the maximal distance tested (14 m), and offers a new option for pheromone-based control strategies. Our recapture rates are comparable to those using other pheromone lures in the field [32,33]. Sufyan et al. [33] had a recapture rate of 70% for click beetles released 2 m away from a point source, which gradually decreased to 40% at 15 m. Hicks and Blackshaw [32] had recapture rates of 75% at 4 m away from a point source, which decreased to 30% at 16 m. The quantity of pheromone used was not reported in either case. Similar to Sufyan et al. [33], we found the beetles’ responses to be immediate, with most recaptures being made within the first three days. However, in contrast with Hicks and Blackshaw [32] and Sufyan et al. [33], the number of recaptures differed between the two release directions. This difference suggests a possible impact of microclimatic conditions on recaptures.

### 4.5. Implications for Pheromone-Based Management Strategies

Whether these rates of recapture are sufficient for economic management of click beetles is largely dependent upon the pheromone application method employed. In an attract-and-kill strategy with autodissemination, only a proportion of the pest population is required to contact the pheromone source in order to afford control. For example, adult German cockroaches (*Blattella germanica* L. (Blattodea: Ectobiidae)) horizontally transfer insecticidal bait to coprophagic nymphs through their feces, which eliminates the need for nymphs to seek and contact the bait [52]. In particular, in a microbial-based autodissemination strategy, infection of a small subset of the population can have a multiplicative impact on population dynamics. Pathogens have the ability to proliferate, and epizootics can occur when a threshold number of the population becomes infected. On the other hand, attract-and-kill strategies that rely on a non-disseminative kill agent may require higher rates of contact.

In our study, female sex pheromone not only induced aggregation of male beetles at the pheromone granules, but also prompted greater activity of males. Possible exploitation of increased mobility prompted by sex pheromone has not been investigated, but increased movement may result in increased pathogen encounter. For example, increased motility in green peach aphids (*Myzus persicae* Sulzer (Hemiptera: Aphididae)), as a result of exposure to alarm pheromone, facilitates increased infection with the fungal pathogen *Verticillium lecanii* Zare and Gams (Hypocreales: Cordycipitaceae) [53]. These findings suggest that increased mobility may be exploited to increase the effectiveness of disseminative insecticides. In addition, there is evidence that increased exercise can affect the physiology of insects, which may have important implications for the use of pheromone with chemical or microbial insecticides. For example, increased activity has been associated with decreased disease resistance in *Gryllus texensis* Cade and Otte (Orthoptera: Gryllidae) crickets [54], and higher immune response in non-foraging bumble bees (*Bombus terrestris* L. (Hymenoptera: Apidae)) [55], which may affect the efficacy of pathogen-based insecticides. Lastly, increased activity may also expose pest species to other predators, which may contribute to the control effort as an indirect effect.

## 5. Conclusions

Beetles respond to female sex pheromone under a range of conditions, and the pheromone appears to be effective throughout the period studied. Lure placement is not expected to be affected by light levels, but it may be most effective to place lures upwind from beetle habitats and reservoirs. Pheromone deployment earlier in the season, however, will increase beetle captures, as they are more active at that time. Much remains to be explored about the internal and external factors that affect the response of *A. obscurus* to sex pheromone in the field, including humidity and temperature, as well as age and prior mating status. Knowledge on how different factors affect pheromone release and beetle response will serve to inform researchers regarding pheromone optimization when considering their use in pest control strategies.

## Figures and Tables

**Table 1:** Model specification. A linear mixed model was used, unless otherwise specified.

Experiment	Response	Transformation	Fixed Effects	Random Effects
Experiment 1: Air movement	Walking speed		Air movement Pheromone Beetle collection	Date of experiment
	Distance walked			
	Time to first contact with granule band	Natural log		
	Number of contacts with granule band	Square-root		
	Duration of contact with granule band	Natural log		
Experiment 2: Light quality	Walking Speed		Light type Pheromone Beetle collection	Date of experiment
	Distance Walked			
	Number of contacts with granules zone	Square-root		
	Duration of contact with granule zone	Natural log		
	Activity duration *			
	Probability of reaching granule zone	Squared		
Experiment 3: Seasonality	Walking speed		Pheromone Beetle collection	Date of experiment
	Distance walked			
	Time spent moving			
Experiment 4: Response range	Proportion of beetles recovered ^		Pheromone Release direction Release distance	Plot

* Generalized linear mixed model with beta distribution. ^ Generalized linear mixed model with binomial distribution and logit link function.

**Table 2:** Statistical model output for the effect of pheromone on the walking speed, distance walked, and proportion of time moving for male *Agriotes obscurus* collected in March, April, and May. Experiment day is included as a random effect.

	Num DF	Den DF	F Value	Pr > F
**Walking speed**				
Pheromone	1	126	32.6	**<0.001**
Beetle collection	2	126	9.64	**<0.001**
Pheromone: Beetle collection	2	124	0.55	0.581
**Distance walked**				
Pheromone	1	126	32.75	**<0.001**
Beetle collection	2	126	8.72	**<0.001**
Pheromone: Beetle collection	2	124	0.47	0.624
**Movement duration**				
Pheromone	1	126	27.87	**<0.001**
Beetle collection	2	126	6.35	**0.002**
Pheromone: Beetle collection	2	124	0.83	0.437

**Table 3:** Mean time (hours ± SE) to maximum recaptures for *Agriotes obscurus* beetles released at different distances away from a pheromone granule band (N = 4).

Release Distance (m)	Mean Hours to Maximum Recapture (±SE)
1	19.3 (4.2)
3	13.1 (0.9)
7	34.6 (2.6)
14	38.1 (8.5)

## Supplementary Materials

The following are available online at https://www.mdpi.com/2075-4450/11/11/729/s1, Table S1: The effect of light (darkness/visible light), beetle collection month and pheromone on male *A. obscurus* walking and activity. Table S2: The effect of the presence of visible light, beetle collection month and pheromone on male *A. obscurus* interaction with pheromone granules. Table S3: LS mean (±SE) walking speed, distance walked, and proportion time moving for male *A. obscurus beetles* in different pheromone-light treatments, for beetles collected in April and May 2014. Table S4: Number of *A. obscurus* beetles used in Experiment 3 to examine the effect of month of collection on activity and response to pheromone
