# Supplementary material for: Effect of Collection Month, Visible Light, and Air Movement on the Attraction of Male Agriotes obscurus L. (Coleoptera: Elateridae) Click Beetles to Female Sex Pheromone
Source: Insects. 2020 Oct 26;11(11):729. doi: 10.3390/insects11110729 (PMC7693911; doi:10.3390/insects11110729)
Supplement: Supplementary file 1 [file insects-11-00729-s001.pdf]

## Supplementary information

**Table S1.** The effect of light (darkness/visible light), beetle collection month and pheromone on male *A. obscurus* walking and activity. A linear mixed model is was conducted and date of experiment was included as a random effect.

| | Num DF | Den DF | F Value | Pr > F |
|---|---|---|---|---|
| **Walking speed** | | | | |
| Pheromone | 1 | 67 | 27.04 | **<0.001** |
| Beetle collection | 1 | 67 | 6.24 | **0.015** |
| Light | 1 | 66 | 0.02 | 0.884 |
| Beetle collection*Pheromone | 1 | 64 | 0.27 | 0.602 |
| Light*Pheromone | 1 | 63 | 0.27 | 0.605 |
| Light*Beetle collection | 1 | 65 | 0.31 | 0.580 |
| Pheromone*Beetle collection*Light | 1 | 62 | 0.96 | 0.331 |
| **Distance walked** | | | | |
| Pheromone | 1 | 67 | 28.52 | **<0.001** |
| Beetle collection | 1 | 67 | 5.62 | **0.025** |
| Light | 1 | 66 | 0.14 | 0.707 |
| Beetle collection*Pheromone | 1 | 64 | 0.04 | 0.836 |
| Light*Pheromone | 1 | 63 | 0.03 | 0.859 |
| Light*Beetle collection | 1 | 65 | 0.51 | 0.476 |
| Pheromone*Beetle collection*Light | 1 | 62 | 1.28 | 0.263 |
| **Proportion time moving** | | | | |
| Pheromone | 1 | 67 | 6.2 | **0.030** |
| Beetle collection | 1 | 67 | 0.58 | 0.449 |
| Light | 1 | 66 | 0.46 | 0.502 |
| Beetle collection*Pheromone | 1 | 63 | 0.00 | 0.983 |
| Light*Pheromone | 1 | 64 | 0.31 | 0.577 |
| Light*Beetle collection | 1 | 65 | 1.21 | 0.276 |
| Pheromone*Beetle collection*Light | 1 | 62 | 0.59 | 0.446 |

**Table S2.** The effect of the presence of visible light, beetle collection month and pheromone on male *A. obscurus* interaction with pheromone granules. A linear mixed model is was conducted unless otherwise stated. Date of experiment was included as a random effect.

| | Num DF | Den DF | F Value | Pr>F |
|---|---|---|---|---|
| **Reach zone*** | | | | |
| Pheromone | 1 | | 8.98 | **0.003** |
| Beetle collection | 1 | | 2.49 | 0.115 |
| Light | 1 | | 0.02 | 0.900 |
| Beetle collection*Pheromone | 1 | | 0.15 | 0.70 |
| Light*Pheromone | 1 | | 1.28 | 0.259 |
| Light*Beetle collection | 1 | | 0.27 | 0.273 |
| Pheromone*Beetle collection*Light | 1 | | 0.052 | 0.820 |
| **Frequency of contacts**+ | | | | |
| Pheromone | 1 | 46 | 6.49 | **0.014** |
| Beetle collection | 1 | 45 | 0.40 | 0.532 |
| Light | 1 | 44 | 0.01 | 0.943 |
| Beetle collection*Pheromone | 1 | 41 | 0.06 | 0.807 |
| Light*Pheromone | 1 | 42 | 0.09 | 0.765 |
| Light*Beetle collection | 1 | 43 | 0.40 | 0.530 |
| Pheromone*Beetle collection*Light | 1 | 40 | 3.07 | 0.088 |
| **Cumulative duration of contact**+ | | | | |
| Pheromone | 1 | 45 | 0.56 | 0.457 |
| Beetle collection | 1 | 46 | 7.47 | **0.009** |
| Light | 1 | 44 | 0.46 | 0.502 |
| Beetle collection*Pheromone | 1 | 42 | 0.29 | 0.596 |
| Light*Pheromone | 1 | 41 | 0.12 | 0.735 |
| Light*Beetle collection | 1 | 43 | 0.69 | 0.410 |
| Pheromone*Beetle collection*Light | 1 | 40 | 1.02 | 0.319 |

* Wald Chi-Square + Analysis only includes beetles that reach granule zone.

**Table S3.** LS mean (±SE) walking speed, distance walked, and proportion time moving for male *A. obscurus beetles* in different pheromone-light treatments, for beetles collected in April and May 2014. The presence of visible light had no effect on measurements and are the data shown are pooled across the light treatments.

| | | **Walking Speed (cms-1)** | **Distance walked (cm)** | **Proportion time moving** |
|---|---|---|---|---|
| April | Blank | 0.84 (0.11) | 470 (67.7) | 0.62(0.12) |
| | Pheromone | 1.37(0.11) | 808 (63.0) | 0.85(0.08) |
| May | Blank | 0.52(0.12) | 304 (72.9) | 0.53(0.13) |
| | Pheromone | 1.14(0.11) | 670 (64.4) | 0.79(0.09) |

**Table S4.** Number of *A. obscurus* beetles used in Experiment 3 to examine the effect of month of collection on activity and response to pheromone.

| | | **Number of groups** | | | **Number of beetles** | | |
|---|---|---|---|---|---|---|---|
| | **Pheromone Treatment** | **17-18 days post collection** | **38-39 days post collection** | **Total** | **17-18 days post collection** | **38-39 days post collection** | **Total** |
| **March** | Pheromone | 12 | 10 | **22** | 60 | 50 | **110** |
| | Blank | 11 | 11 | **22** | 55 | 55 | **110** |
| **April** | Pheromone | 13 | 12 | **25** | 65 | 35 | **100** |
| | Blank | 11 | 11 | **22** | 54 | 34 | **88** |
| **May** | Pheromone | 12 | 12 | **24** | 60 | 40 | **100** |
| | Blank | 12 | 12 | **24** | 60 | 35 | **95** |
